# SweetBac: A New Approach for the Production of Mammalianised Glycoproteins in Insect Cells

**DOI:** 10.1371/journal.pone.0034226

**Published:** 2012-04-02

**Authors:** Dieter Palmberger, Iain B. H. Wilson, Imre Berger, Reingard Grabherr, Dubravko Rendic

**Affiliations:** 1 Vienna Institute of BioTechnology - VIBT, University of Natural Resources and Life Sciences, Vienna, Austria; 2 European Molecular Biology Laboratory - EMBL, Grenoble, France; Central China Normal University, China

## Abstract

Recombinant production of therapeutically active proteins has become a central focus of contemporary life science research. These proteins are often produced in mammalian cells, in order to obtain products with post-translational modifications similar to their natural counterparts. However, in cases where a fast and flexible system for recombinant production of proteins is needed, the use of mammalian cells is limited. The baculoviral insect cell system has proven to be a powerful alternative for the expression of a wide range of recombinant proteins in short time frames. The major drawback of baculoviral systems lies in the inability to perform mammalian-like glycosylation required for the production of therapeutic glycoproteins. In this study we integrated sequences encoding *Caenorhabditis elegans N*-acetylglucosaminyltransferase II and bovine β1,4-galactosyltransferase I into the backbone of a baculovirus genome. The thereby generated SweetBac virus was subsequently used for the production of the human HIV anti-gp41 antibody 3D6 by integrating heavy and light chain open reading frames into the SweetBac genome. The parallel expression of target genes and glycosyltransferases reduced the yield of secreted antibody. However, the overall expression rate, especially in the recently established *Tnao*38 cell line, was comparable to that of transient expression in mammalian cells. In order to evaluate the ability of SweetBac to generate mammalian-like N-glycan structures on 3D6 antibody, we performed SDS-PAGE and tested for the presence of terminal galactose using *Riccinus communis* agglutinin I. The mammalianised variants of 3D6 showed highly specific binding to the lectin, indicating proper functionality. To confirm these results, PNGase A released N-glycans were analyzed by MALDI-TOF-MS and shown to contain structures with mainly one or two terminal galactose residues. Since the presence of specific N-glycans has an impact on antibodies ability to exert different effector functions, we tested the binding to human Fc gamma receptor I present on U937 cells.

## Introduction

The use of insect cells for the production of therapeutically active proteins has gained increasing importance over the last decade, highlighted by the market entry of the human papilloma virus vaccine Cervarix™ [1]. Nowadays, a set of different insect cell lines, suitable for the baculovirus-driven production of recombinant proteins, is available. The most popular ones are *Spodoptera frugiperda Sf*9 cells [2], *Spodoptera frugiperda Sf*21 cells [3] and *Trichoplusia ni* BTI-TN5B1-4 “High Five” (“Hi5”) cells [4]. More recently, a new *Trichoplusia ni* cell line, BTI-*Tnao*38, has been established [5]. A major limitation regarding the production of therapeutic proteins in insect cell lines is the lack of complex type N-glycans, often leading to a severely reduced efficacy. N-glycans found on insect cell expressed proteins are mainly of a high mannose type or non-fucosylated and core-fucosylated tri-mannose structures [6].

In the past, several approaches have served to improve N-linked glycosylation in lepidopteran insect cell lines by expressing additional glycosyltransferases. In a first trial, *Sf*9 cells were co-infected with standard baculovirus vectors expressing human β1,2-*N*-acetylglucosaminyltraferase I (GnTI) and a fowl plague virus haemagglutinin (HA). Analysis of HA glycosylation patterns gave a four fold increase in the amount of terminal *N*-acetylglucosamine (GlcNAc) indicating the proper functionality of human GnTI in insect cells [7]. In another approach, the use of a recombinant baculovirus expressing a bovine β1,4-galactosyltransferase driven by the immediate early promoter 1 (IE1) was investigated. It could be shown that the baculovirus envelope protein gp64 was modified by an additional galactose on at least one N-linked oligosaccharide side chain [8]. These strategies are based on transient expression of glycosyltransferases. Other studies have evaluated the use of stable integration for modifying insects N-linked glycosylation pathways. Hollister et al (1998) constructed a plasmid expressing a bovine β1,4-galactosyltransferase controlled by the baculoviral IE1 promoter and stably integrated it into the genome of *Sf*9 cells. A clone that has been selected for high enzymatic activity produced a galactosylated “human tissue plasminogen activator” protein after infection with the recombinant baculovirus [9]. The production of terminally sialylated biantennary N-glycans was the next step in glycoengineering insect cells. Several studies have shown that the stable integration of β1,4-galactosyltransferase and α2,6-sialylltransferase leads to partially sialylated gp64 after infection with the corresponding recombinant baculovirus [10], [11]. To further extend the N-glycan processing, a set of five mammalian glycosyltransferases was integrated into the genome of *Sf*9 cells [12]. Due to the fact that the intracellular levels of sialic acid and CMP-sialic acid, necessary for proper sialylation, are very low in insect cells, a new transgenic *Sf*9 insect cell line was generated. This was achieved by the additional integration of genes encoding sialic acid synthetase and CMP-sialic acid synthetase leading to a total number of 7 constitutively expressed glycozymes [13]#. The drawback with using such a setup is a possible metabolic overload for the transgenic cell line, leading to reduced growth characteristics and long-term stability as well as reduced yields of recombinantly produced proteins. Furthermore, the altered glycosylation pattern might influence the functionality of cellular proteins and have a wider impact on the robustness of the system. To overcome these problems, a recent study has reported the generation of a new transgenic *Sf*9 cell line with an inducible mammalian-like protein N-glycosylation machinery [14]. What still remains is the fact that all these approaches are based on *Sf*9 cells. However, for the expression of secreted proteins, cell lines derived from *Trichoplusia ni* have been demonstrated to produce significantly higher amounts in many cases [15], [16]. Therefore, we decided to revive the idea of using the baculovirus itself for glycoengineering purposes. The MultiBac platform, which is an advanced version of the Bac-to-Bac system, provides the possibility of flexible multigene expression using a single baculovirus vector [17]–[20]. In this study we evaluated the use of MultiBac for the generation of an efficient platform for the production of properly glycosylated proteins in lepitopteran insect cells other than the commonly used *Sf*9 cells.

## Materials and Methods

All DNA manipulations were carried out essentially as summarised by Sambrook *et al.* [21]#. Restriction enzymes, T4 DNA ligase and Calf Intestinal Alkaline Phosphatase were purchased from New England Biolabs (Ipswich, USA). DNA polymerase was purchased from Novagen (Darmstadt, Germany). All enzymes were used according to manufacturer's recommendation. All primers and oligos were synthesised by Sigma-Aldrich (St. Louis, USA).

### Cells and viruses

*Spodoptera frugiperda Sf*9 cells (ATCC CRL-1711) [2], *Trichoplusia ni* BTI-TN5B1-4 “High Five” (“Hi5”) cells (ATCC CRL-10859) [4] and *Trichoplusia ni* BTI-*Tnao*38 cells [5] were grown in IPL-41 medium (SAFC Biosciences, St. Louis, USA) containing yeast extract, a lipid mixture supplemented with either 0% or 3% fetal calf serum (FCS) at 27°C using T-flasks.

Human U937 leukemic monocyte lymphoma cells (ATCC CRL-1593.2) were cultivated in RPMI1640 medium (PAA, Pasching, Austria) containing 10% FCS and 4 mM glutamine at 37°C using T-flasks. Recombinant *Autographa californica* nucleopolyhedroviruses were isolated and plaque purified by standard procedures. Viral titres were determined by plaque assay using 10-fold dilution series (n = 3).

### Construction of SweetBac

The *Caenorhabditis elegans N*-acetylglucosaminyltransferase II (GnTII) was PCR-amplified from a plasmid containing full length open reading frame (ORF) of the GnTII using primers CeGnTII-for (5′- GAC TTG ATC ACC CGG GAT GAT GGT CTA TCG ACG GAT G -3′) and CeGnTII-rev (5′- TGC ATC AGC TGC TAG CTT AAG AAG TTG TAG ATG TGA TTG T -3′). The product was digested with NheI/XmaI and ligated into pUCDM vector (EMBL, Grenoble) cut with same enzymes, resulting in pUCDM-G. The bovine ß1,4-galactosyltransferase I (GalT) was PCR amplified from a plasmid containing full length ORF of the GalT using primers b4GalT-for (5′- CAT CGG GCG CGG ATC CAT GAA GTT TCG GGA GCC GCT -3′) and b4GalT-rev (5′- GAC TGC AGG CTC TAG ACT AGC TCG GCG TCC CGA TG -3′). The product was digested with BamHI/XbaI and ligated into pUCDM-G vector cut with the same enzymes resulting in pUCDM-GG. The resulting glyco-module was introduced in the loxP site of a MultiBac genome, using DH10MultiBac^Cre^ cells according to Fitzgerald et al. [18]. The hence generated DH10MultiBac^Glyco^ cells were then used as a basis for the generation of a recombinant baculovirus expressing an IgG_1_ antibody.

### Construction of SweetBac expressing human IgG_1_ heavy and light chain genes

The human HIV anti-gp41 IgG_1_ antibody 3D6 [22] was used as a model protein. The antibody light chain was PCR amplified using primers LC-SpeI-for (5′- AGTAGTAGTACTAGTATGGACATGAGGGTCCCCG -3′) and LC-HindIII-rev (5′- AGTAGTAGTAAGCTTCTAACACTCTCCCCTGTTGAAGC -3′). The product was digested with SpeI/HindIII and ligated into pFastBac Dual (Invitrogen, Carlsbad, USA) vector cut with same enzymes, resulting in pFBD-LC. The dsDNA encoding 3D6 heavy chain was PCR amplified using primers HC-XhoI-for (5′- AGTAGTAGTCTCGAGATGGAGTTGGGACTGAGCTG -3′) and HC-NheI-rev (5′- AGTAGTAGTGCTAGCTCATTTACCCGGAGACAGGG -3′). The product was XhoI/NheI digested and ligated into XhoI/NheI digested pFBD-LC, resulting in pFBD-3D6. The pFBD-3D6 plasmid was inserted into the Tn7 site of the previously generated SweetBac (see “Construction of SweetBac” section above) using DH10MultiBac^Glyco^ cells allowing generation of SweetBac-3D6 according to standard procedures. As a negative control, the pFBD-3D6 plasmid was inserted in a “wild-type” bacmid using DH10MultiBac cells followed by generation of virus according to standard procedures.

### Expression and purification of mAb 3D6

*Tnao*38 and Hi5 cells were transferred to shaker flasks with supplemented IP-L41 medium without FCS two days prior to infection. For infection, 5×10^7^ cells were spun down at 900× g for 10 min, resuspended in 50 mL fresh IP-L41 medium and infected with a multiplicity of infection (MOI) of 5. All expressions were carried out in triplicate. To generate an expression profile, samples were taken from 24 to 168 hours post infection (hpi) every 24 hours. For standardisation of further experiments, supernatants of *Tnao*38 and Hi5 cells were harvested 96 hpi by centrifugation at 2000 g for 10 min.

The cellular supernatant was filtered through a 0.22 µM filter cartridge (Millipore, Billerica, USA) and applied on a 1 mL Bio-Scale™ Mini UNOsphere SUPrA™ Cartridge (BioRad, Hercules, USA) using an Äkta purifier (GEHealthcare). Elution was performed with 100 mM glycine/HCl buffer pH 3. To increase stability, the eluted samples were adjusted to pH 7 using 100 mM Tris-HCl (pH 9).

### Enzyme-linked Immunosorbent Assay

Coating of 96 well Maxisorp plates (Nunc, Roskilde, Denmark) was performed by incubating 100 µL anti-Human IgG (γ-chain specific) antibody (Sigma Aldrich, I3382) (diluted 1∶1000 in coating buffer: PBS, 1% BSA) at 4°C over night. Plates were washed with TPBS (PBS, 0.1% Tween20) and 50 µL serially diluted samples (in dilution buffer: TPBS, 1% BSA) were applied to the wells. After one hour incubation on a rocking platform, the plates were washed again with TPBS. Detection was performed with 50 µL/well of an anti-human κ light chain peroxidase conjugate (Sigma Aldrich, A7164) 1∶1000 diluted in dilution buffer for 1 h while rocking. Staining of the ELISA plates was performed by adding 100 µL/well staining buffer (sodium citrate buffer, pH5) containing 1 mg/mL OPD (1,2-*o*-phenylenedihydrochloride) and 1 µL/mL H_2_O_2_ (35%). The reaction was stopped with 100 µL H_2_SO_4_ and plates were analysed on an Infinite® M1000 reader (Tecan Group, Männedorf, Switzerland).

### SDS-PAGE and lectin blotting

Samples were mixed with 2× electrophoresis buffer containing 250 mM Tris/HCl, pH 6.8, 10% glycerol, 2% SDS, 100 mM DTT and 0.1% bromophenol blue. Proteins were separated by SDS-Page according to Laemmli [23]#, stained with colloidal Coomassie or electroblotted onto a nitrocellulose membrane (Pall, Port Washington, USA). Detection was performed using biotinylated *Ricinus communis* agglutinin I (RCA I) (Vector Labs, B-1085) in TPBS (PBS+0.05% Tween 20) and streptavidin-conjugated alkaline phosphatase (Vector-Labs, SA-5100). The blot was developed using NBT/BCIP Sigma fast tablets (Sigma, B5655).

### N-glycan analysis

N-glycan analyses were essentially performed as described in Rendic et al. [24]. Briefly, after the heavy and light chains of purified IgG_1_ were separated on an SDS-PAGE, bands corresponding to heavy chains were excised. After washing the bands, they were treated with dithiothreitol and iodoacetamide solutions to modify cysteine residues. The gel bands were washed again and then subjected to trypsin digestion at 37°C over night. The peptides extracted with AcN∶H_2_O∶TFA solution (acetonitrile/water/trifluoroacetic acid; 666∶333∶1) were then dried, resuspended in 20 µl of 50 mM ammonium acetate (pH 5) buffer and subjected to PNGase A treatment at 37°C over night. The released N-glycans were separated from peptides by using columns packed with 10 µL LiChroprep RP 18 (25–40 µM) reversed phase resin (Merck) on top of 40 µL Dowex 50WX8-400 ion exchange resin (Sigma, 217514). After equilibrating the column with 2% acetic acid, the sample was applied, the column was washed with 2% acetic acid and the flow-through containing N-glycans was collected. For further purification of the released N-glycans, columns were packed with Supelclean™ ENVI-Carb™ PGC material (Sigma) on top of LiChroprep RP 18 (25–40 µM) reversed phase resin (Merck) and equilibrated with 2% acetic acid. The samples were loaded on the column and after washing with H_2_O, the N-glycans were eluted with 40% acetonitrile. The purified N-glycans were dried in a SpeedVac, finally resuspended in 5 µL deionised water and used for MALDI-TOF-MS analysis (on a Bruker Ultraflex MALDI-TOF/TOF in positive reflectron mode) using 6-aza-2-thiothymine (ATT) as matrix.

### Flow cytometric analysis

*Sf*9 cells were infected with a recombinant baculovirus expressing the HIV gp41 3D6 epitope (SGKLICTTAVPWNAS) on the cellular surface with MOI 10 and harvested 72 hpi. After brief washing with phosphate buffered saline (PBS), 1×10^6^ cells were incubated with 100 µL of mammalianised/“wild-type” 3D6 antibody (1 µg/mL in PBS containing 10% FCS) for 1 h at room temperature while gently shaking. Cells were again washed with PBS and incubated with an anti-human IgG (γ-chain specific) R-phycoerythrin conjugate (Sigma-Aldrich, P8047) in PBS containing 10% FCS for 1 h at room temperature. After a final wash with PBS, cells were resuspended in 400 µL PBS and analysed on a FACS-CantoII (Becton Dickinson, San Jose, CA).

Human U937 leukemic monocyte lymphoma cells were washed with PBS and incubated with 100 µL of mammalianised/“wild-type” 3D6 antibody (1 µg/mL in PBS) for 1 h at room temperature while gently shaking. After a brief washing, cells were incubated with an anti-human κ light chain FITC conjugate (Sigma Aldrich, F3761). Cells were finally washed with PBS, resuspended in 400 µL PBS and analysed on a FACS-CantoII.

## Results

### Construction of SweetBac

**Figure 1:**
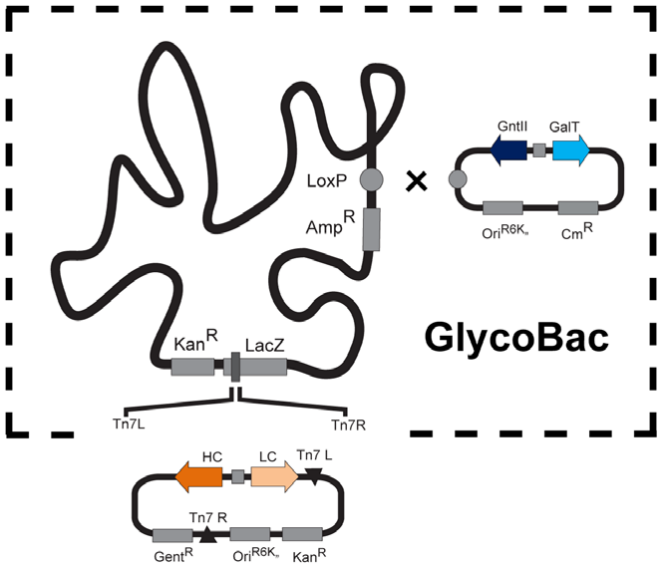
Schematic representation of SweetBac. A glyco-module, consisting of the open reading frames of the *C. elegans N*-acetylglucosaminyltransferase II (GnTII) and the bovine β4-galactosyltransferase I (GalT), was integrated in the loxP site of a MultiBac genome resulting in SweetBac. The generated viral backbone was subsequently used for the expression of 3D6 antibody heavy (HC) and light (LC) chain genes, by integrating them in the standard Tn7 site.

Our aim was to develop a virus backbone, facilitating flexible modifications of the N-linked glycosylation capacity of *Sf*9, *Sf*21, Hi5 and *Tnao*38 insect cells. Previous results have shown that mammalian β1,4-galactosyltransferase I (GalT) can efficiently modify the glycosylation of insect cells [9]#. Our own data further indicate that the invertebrate GLY-20 *N*-acetylglucosaminyltransferase II (GnTII) of *C. elegans*, whose temperature optimum is near to that where insect cells are grown, was well expressed in *Sf*9 cells (Wang H., Rendic D., unpublished data). Therefore, we constructed a glyco-module coding for a *C. elegans* GnTII and a bovine GalT controlled by p10 and polyhedrin (ph) promoter respectively. This module was integrated into the loxP site of a MultiBac genome. The resulting SweetBac was then used as a basis for the production of recombinant glycoproteins with mammalianised complex type N-glycans. In order to evaluate the functionality of SweetBac, we additionally introduced heavy (HC) and light chain (LC) open reading frames of the human HIV anti-gp41 antibody 3D6 [22] in the Tn7 site of a SweetBac genome, resulting in SweetBac-3D6. As a negative control, open reading frames coding the heavy and the light chains of the same antibody were integrated in a “wild-type” MultiBac genome as well (Figure 1).

### Expression levels of 3D6 antibody in Hi5 and *Tnao*38 cells

**Figure 2:**
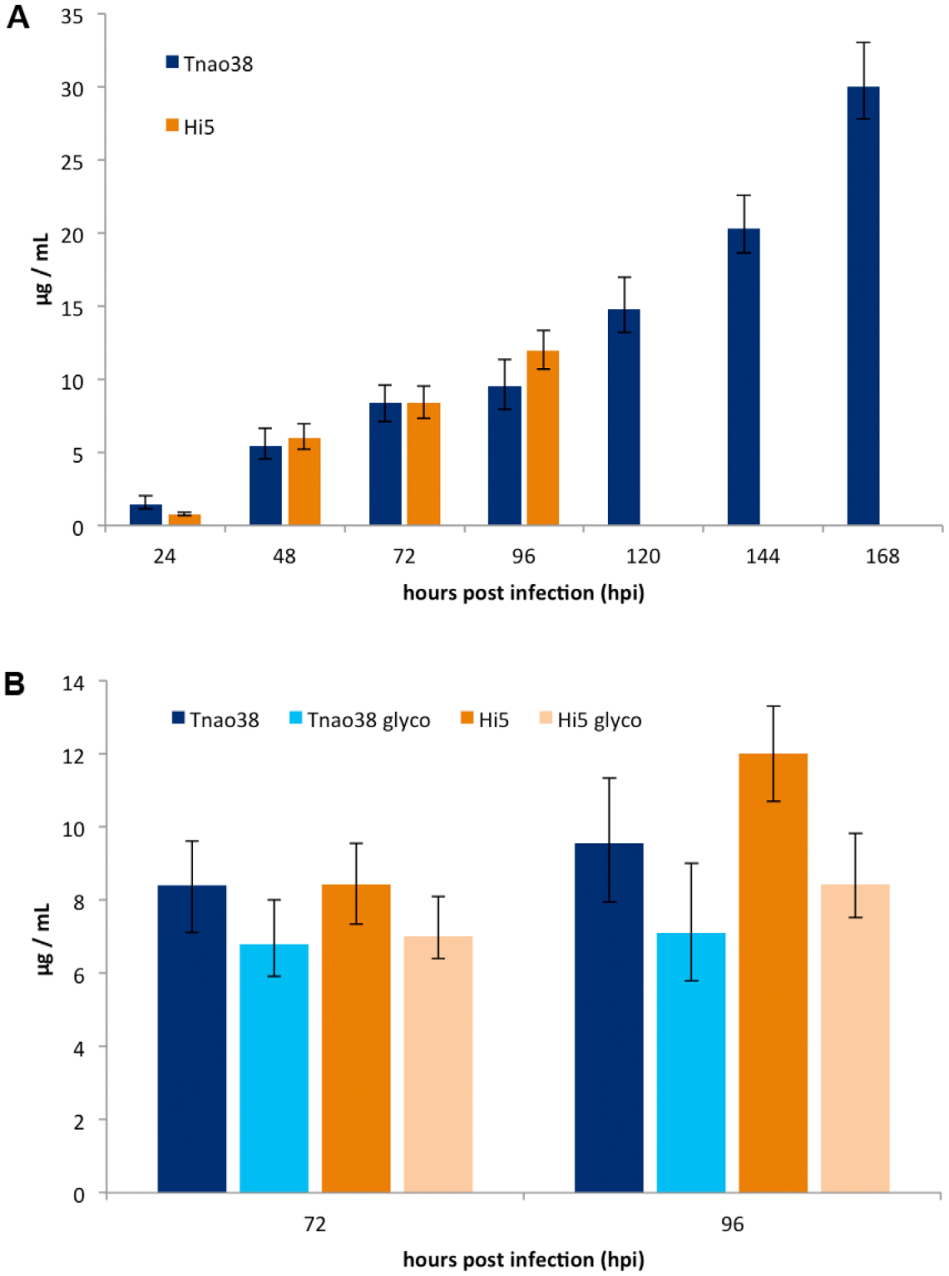
Expression of 3D6 antibody in lepidopteran insect cells. (A) *Tnao*38 and Hi5 cells were infected in triplicate with recombinant baculovirus expressing 3D6 antibody. Samples were taken every 24 h and the amount of secreted antibody was measured by ELISA. (B) *Tnao*38 and Hi5 cells were again infected in triplicate with recombinant baculovirus (Tnao38, Hi5) and SweetBac virus (*Tnao*38 glyco, Hi5 glyco) expressing 3D6 antibody. Samples were taken 72 and 96 hours post infection (hpi) and the amount of secreted antibody was measured by ELISA. Significance of the results was confirmed by Student's t-test (*p*-value<0,05).

Hi5 and *Tnao*38 cells are both derived from *Trichoplusia ni*. Previous studies have demonstrated higher yields, especially for secreted proteins, when using Hi5 cells [15]. In order to evaluate antibody expression yields for both cell lines, we infected *Tnao*38 and Hi5 cells with a recombinant baculovirus expressing 3D6 antibody at an MOI of 5 and took samples every 24 hours up to 4 or 7 days; Hi5 cells started to lyse after 4 days and thus, were not cultivated any longer. The amount of secreted antibody was measured by ELISA. Figure 2A shows that the secretion profile among the two cell lines was similar within the first 96 hours post infection (hpi). *Tnao*38 and Hi5 cells yielded around 1 µg of secreted antibody/ml cell culture supernatant 24 hpi, steadily increasing to 9.5 and 12 µg respectively at 96 hpi. *Tnao*38 cells were more stable, allowing the expression to be expanded up to 168 hpi. A continuous increase in antibody secretion could be detected, yielding up to 30 µg/ml on day 7. These data indicate that *Tnao*38 cells are even more robust than Hi5 cells and suggest them to be, at least for this antibody, highly suitable for large scale protein production.

To evaluate whether co-expression of the glycosyltransferases has an impact on target gene expressions, we infected *Tnao*38 and Hi5 cells with a SweetBac virus expressing the 3D6 antibody (SweetBac-3D6) and with standard baculovirus expressing 3D6. Samples were analysed at 72 hpi and 96 hpi for both cell lines. Figure 2B shows that at 72 hpi, the amounts of secreted 3D6 antibody from *Tnao*38 and Hi5 cells decreased 18% as compared to 3D6 expression without glycosyltransferase genes in the baculovirus genome. At 96 hpi, the yields were 25% lower for *Tnao*38 cells and 30% lower for Hi5 cells when SweetBac-3D6 was used. Significance of the results was confirmed by Student's t-test (*p*-value<0.05).

### Testing the glycosylation of 3D6

**Figure 3:**
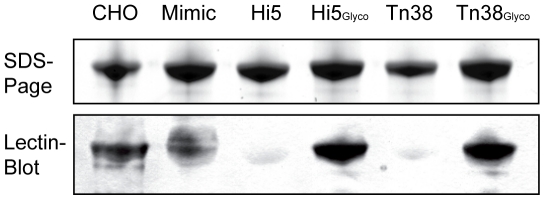
Lectin Blot using *Ricinus communis* agglutinin I. Cellular supernatants were harvested 96 hpi and purified 3D6 antibodies were separated on SDS-PAGE and binding of biotinylated *Riccinus communis* agglutinin I to antibody heavy chains was tested. The lectin only bound SweetBac expressed antibodies (Hi5Glyco, Tn38Glyco), because of terminal galactose present on N-glycans. CHO and Mimic insect cell expressed antibodies were used as controls.

For testing the functionality of SweetBac, purified 3D6 antibodies were subjected to SDS-PAGE and electroblotted to a nitrocellulose membrane. The presence of galactose residues was detected by incubating the blot with *Ricinus communis* agglutinin I (RCA I), specific for terminal galactose [25]#. Figure 3 shows that although similar protein amounts of antibody heavy chains were loaded on the gel, RCA I bound to only mammalianised variants (HiGlyco and Tn38Glyco). These results confirmed the desired change in glycosylation, indicating proper functionality of the integrated glycosyltransferases. 3D6 derived from Chinese hamster ovary cells (CHO) as well as one derived from Mimic *Sf*9 cells, a cell line containing a human *N*-acetylglucosaminyltransferase I, human *N*-acetylglucosaminyltransferase II, rat β1,4-galactosyltransferase I, mouse α2,3-sialyltransferase and mouse α2,6-sialyltransferase [12] served as controls.

### N-glycan analysis

**Figure 4:**
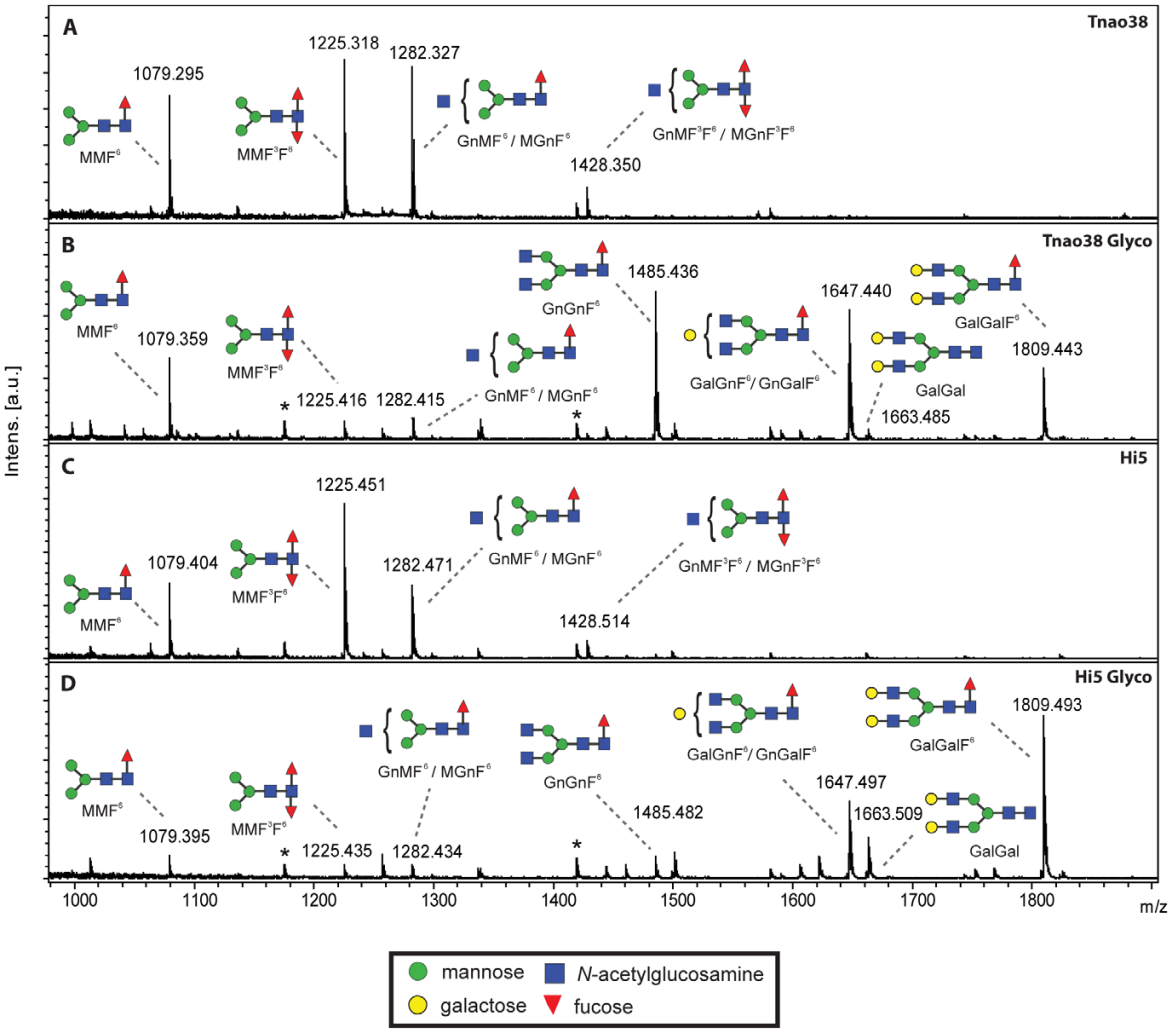
MALDI-TOF-MS spectra of 3D6 IgG_1_ N-glycans. N-glycan structures of an IgG_1_ antibody expressed in *Tnao*38 (A) and Hi5 (C) cells (96 hpi) mostly consist of single and double fucosylated tri-mannose structures with maximally one terminal *N*-acetylglucosamine. The spectra of the mammalianised cells (B, D) show a shift of the dominant structures towards higher m/z values. Fucosylated biantennary N-glycan structures carrying two *N*-acetylglucosamine residues with one or two terminal galactose residues were identified as dominant N-glycan on IgG_1_ recombinantly expressed in *Tnao*38 cells using SweetBac (B). The N-glycan structures identified from IgG_1_ expressed in Hi5 cells using SweetBac (D) resemble the ones found in SweetBac infected *Tnao*38 cells with an even higher dominance of terminal galactose. Oligomannosidic glycans (Man_5–6_GlcNAc_2_) are indicated by asterisks. Graphical representations of glycans are consistent with the nomenclature of the Consortium for Functional Glycomics.

In order to confirm the results obtained with lectin blotting, we analyzed the N-glycosylation patterns of the different 3D6 antibodies by mass spectrometry. Figure 4 shows the MALDI-TOF-MS spectra of PNGase A released IgG_1_ N-glycans from mammalianised and “wild-type” *Tnao*38 and Hi5 cells. The 3D6 expressed in *Tnao*38 cells carries mainly single and double fucosylated tri-mannose structures, partly with one terminal N-acetylglucosamine. 3D6 antibodies from mammalianised *Tnao*38 cells on the other hand showed mainly complex, singly fucosylated biantennary N-glycan structures carrying two terminal *N*-acetylglucosamine residues (∼30%), one terminal galactose (∼30%) or two terminal galactose residues (∼20%). N-glycans from Hi5 cell expressed 3D6 closely resemble those found from *Tnao*38 cells, but are more highly galactosylated. The portion of complex biantennary structures was as high as ∼80% from mammalianised variants with ∼50% carrying two terminal galactose residues.

### Functionality of 3D6

**Figure 5:**
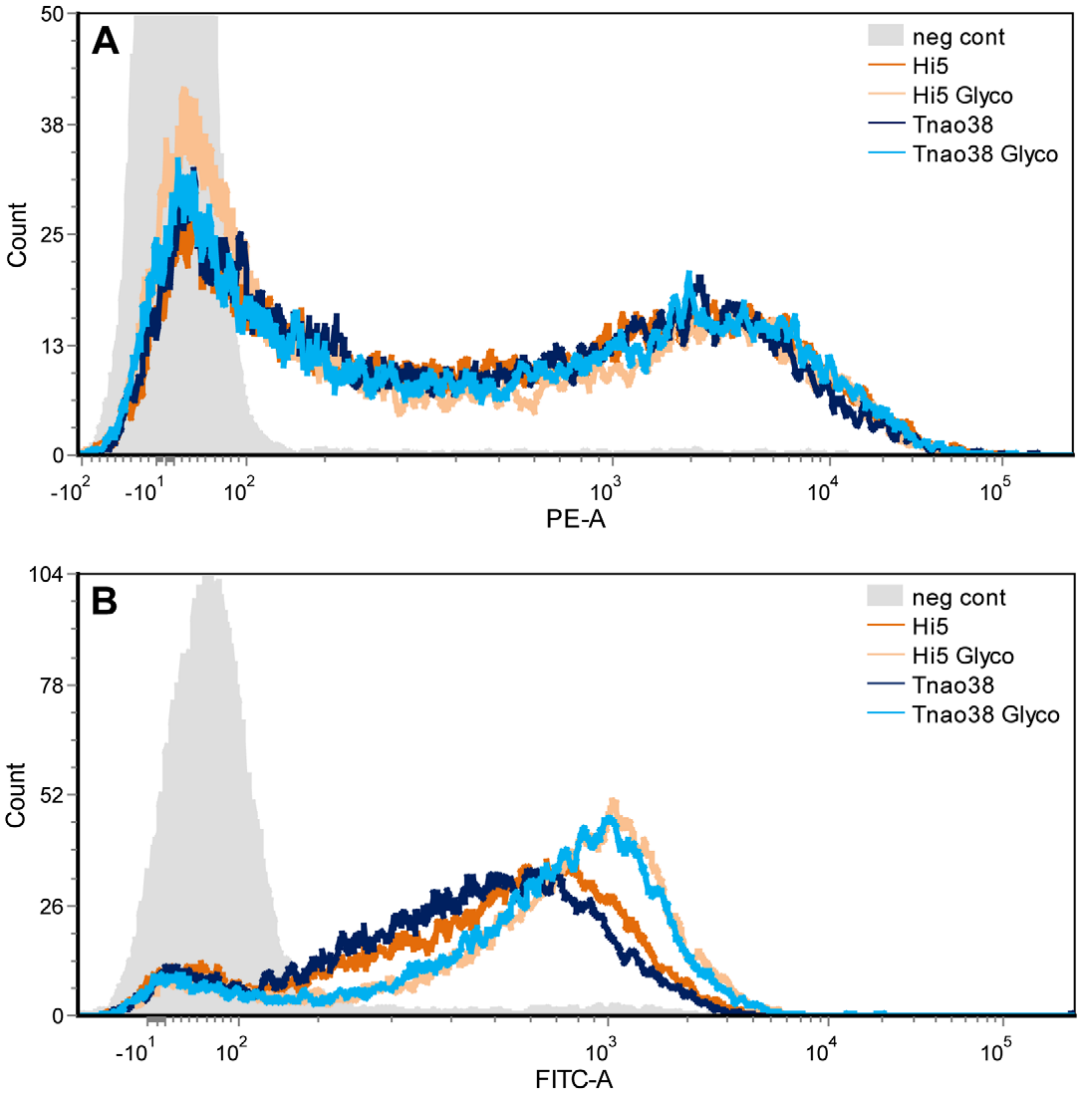
Binding of insect cell expressed 3D6 antibodies to 3D6 epitope and human FcγRI. *Sf*9 cells presenting the 3D6 epitope were incubated with purified antibodies and target binding was analysed by FACS. Antibodies expressed in Hi5 and *Tnao*38 cells using standard baculoviral vectors (Hi5, *Tnao*38) as well as SweetBac (Hi5 Glyco, *Tnao*38 Glyco) show a highly specific binding. The broadening of the peaks can be explained by different amounts of 3D6 epitope displayed on *Sf*9 cells (A). Binding of antibodies to human Fc gamma receptor I was measured by incubating 3D6 antibodies with U937 cells. FACS analysis showed that all antibodies bound FcγRI present on the cellular surface, but the binding of SweetBac expressed variants (Hi5 Glyco, *Tnao*38 Glyco) was significantly increased.

In order to evaluate the target binding ability of the different 3D6 antibodies, we infected *Sf*9 cells with a recombinant baculovirus expressing the HIV gp41 epitope (SGKLICTTAVPWNAS) on the cellular surface. The epitope-presenting cells were incubated with 3D6 antibody and analysed by FACS. Figure 5A shows that the binding characteristics were equal for all antibody preparations, indicating the same specificity and affinity.

Besides target binding, the ability of an antibody to elicit glycosylation dependent effector functions was evaluated. Human U937 leukemic monocyte lymphoma cells that are known to express the human Fc gamma receptor I (CD64) on their cellular surface were incubated with 3D6 antibody samples and receptor binding was again analysed by FACS. Figure 5B shows that binding characteristics of antibodies produced in Hi5 and *Tnao*38 cells were comparable. However, the peaks resulting from samples derived from SweetBac-3D6 infected cell lines are shifted in both cases. This increase in binding capacity indicates the importance of proper, mammalian-like N-glycosylation for recombinantly expressed antibodies [26]–[28].

## Discussion

The presence of specific N-linked glycan structures is of great importance for the full functionality of therapeutically active proteins. Mammalian cells, especially Chinese hamster ovary (CHO), human embryonic kidney (HEK) and mouse myeloma (NS0) cells, are currently the favoured cell lines for the production of functional proteins like antibodies, because of high yields and their ability to generate complex type N-glycans [29], [30]. A major drawback in the field of mammalian cell technology is the either time consuming top-clone selection for generating transgenic cell lines, the labour-intensive transfection procedures for transient expressions or, occasionally, the occurrence of allergenic α1,3-galactose [31]. The baculovirus-insect cell system may have an advantage over stably transfected CHO cells, when considering a need for fast and easy production systems which may get increasingly important in the future due to novel, “personalised” medical treatments or when time consuming production schemes are not acceptable.

Based on data proposing that Hi5 cells are more suitable for expressing high amounts of secreted proteins as compared to the commonly used *Sf*9 cells [15], [16], we chose to evaluate the expression capacity of Hi5 cells and the newly established *Tnao*38 cell line [5]#. Using *Tnao*38 cells, the expression could be expanded up to 7 days post infection because of very high cellular stability. In the end, the amount of secreted 3D6 antibody was as high as 30 µg/mL cell culture supernatant, which is approaching production levels of transient expressions in mammalian cells (Figure 2). The higher robustness of this cell line might be an attractive feature for up-scaling the production system. The less virulent infectious cycle is also reflected by 1–2 magnitudes lower baculovirus titres in the supernatant as compared to *Sf*9 cells (data not shown). The lower baculovirus titres in the supernatant are expected to reduce issues in downstream processing facilitating quicker and more effective steps towards acquiring pure (and virus-free) recombinantly produced protein.

In this study we further evaluated the possibility to mammalianise the insect glycosylation machinery by a viral-based approach in order to provide a flexible protein production platform suitable for various insect cell lines. The differences in N-glycan processing between insect and mammalian cells are due mainly to insignificant expression of glycosyltransferases responsible for generating complex-type structures. Glycoengineering in insect cells is based mainly on the pioneering work of Jarvis and co-workers and is generally performed by stably integrating enzymes into the cellular genome of *Sf*9 cells [12], [13]. This, however, is a very time consuming process and a flexible choice of the most suitable cell line is lost. In a previous study we have furthermore shown that *Sf*9 cells are not always the ideal choice when it comes to the production of secreted proteins in high yields [16]#.

Our approach to provide a more flexible glycosylation to a wide range of insect cell lines is based on the MultiBac technology [17]–[20] which is feasible for the expression of multiple genes from one baculovirus backbone. We integrated the *C. elegans N*-acetylglucosaminyltransferase II (GnTII) and the bovine β1,4-galactosyltransferase I (GalT) into the backbone of a standard MultiBac virus, resulting in a recombinant baculovirus designated as SweetBac. We further inserted heavy and light chain genes of the human HIV anti-gp41 IgG_1_ antibody 3D6 [22] as a model, resulting in the baculovirus vector SweetBac-3D6. A great advantage of this setup is that the enzymes necessary for glycosylation as well as the genes of interest are present on one single baculoviral vector. Co-expression of proteins in one cell by co-infection of two or more recombinant baculovirus clones is generally possible. However, the use of multiple baculovirus clones for co-infection decreases the chances that all required genes are delivered to all cells in the population.

Studies have already reported the expression of glyco-modifying enzymes and target protein from the same viral vector, however, integration was based on a quadruple transfer-vector with a size of 16.6 kb [32]#. This approach limits the possibility of integrating further enzymes and/or target proteins. The reason for initially focussing on two glycosyltransferases was the fact that proper sialylation requires a whole set of enzymes and thus, is beyond the scope of demonstrating the proof of concept [13]. However, the existing SweetBac virus may further be modified by integrating respective expression cassettes into its backbone.

When SweetBac-3D6 was used to infect Hi5 and *Tnao*38 cells the yield of antibody was significantly reduced as compared to expression without a glycosylation module. This might be due to the very strong promoters used for the expression of glycosyltransferases. Using the weak immediate early promoter IE1, providing proper glycosylation earlier during the infection cycle, might have a positive impact on the product as well.

In order to evaluate the actual change in glycosylation of 3D6, we performed a lectin blot detecting terminal galactose. Figure 3 shows that specific binding was only detected with mammalianised antibodies indicating a proper functionality of the introduced glycosyltransferases. As a conformation, we analysed PNGase A released N-glycans by MALDI-TOF-MS. Indeed, SweetBac-3D6 derived antibody carries complex biantennary N-glycans, mainly with one or two terminal galactose residues. Nevertheless, the amount of galactosylated glycans on IgGs expressed in mammalianised Hi5 cells is significantly higher (∼50% compared to ∼20% in *Tnao*38 cells) (Figure 4). In terms of the degree of galactosylation, SweetBac mammalianised Tnao38 cells produced IgG akin to that observed for CHO cells [16]. However, Tnao38 cell derived IgGs also carried more paucimannosidic structures, lacking terminal GlcNAc or galactose; these types of glycans are only present at low levels on IgGs expressed by Hi5 cells and are absent from CHO cells.

It is known that the N-linked glycosylation of an IgG_1_ antibody strongly influences the ability to exert effector functions like antibody-dependent cellular cytotoxicity (ADCC). We therefore investigated the binding of 3D6 to human Fcγ receptors present on human U937 leukemic monocyte lymphoma cells, as well as the binding to its target. The presence of a more complex N-glycan structure did not influence target binding (Figure 5A), but binding to Fc receptors was significantly enhanced (Figure 5B), indicating that one can improve the efficacy of insect cell expressed antibodies by co-expressing two key glycosyltransferases using the SweetBac system. As insect cell expression system has been gaining more importance during the past years [1], and new cell lines are being established [5], we aim to provide a flexible tool for production of custom-designed individual products at sufficient yields and within a reasonable time frame.
