# Correction: SweetBac: A New Approach for the Production of Mammalianised Glycoproteins in Insect Cells

**DOI:** 10.1371/annotation/bd906c17-a44b-443d-9051-7d31d8e47afa

**Published:** 2012-08-08

**Authors:** Dieter Palmberger, Iain B. H. Wilson, Imre Berger, Reingard Grabherr, Dubravko Rendic

There is an error in Figure 1. The revised, correct Figure 1 can be seen here: 

**Figure:**
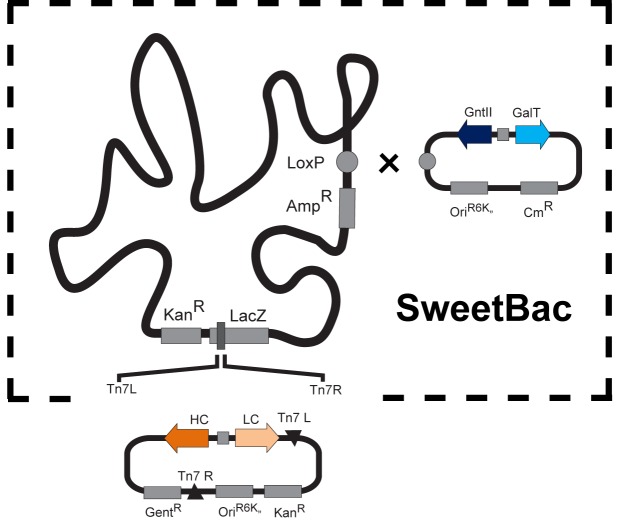


[^] 

